# Periodization, training intensity distribution, and blood lactate monitoring in a female Olympic champion 20 km race walker: a longitudinal case study

**DOI:** 10.3389/fspor.2026.1893820

**Published:** 2026-08-04

**Authors:** Qiuyan Jiang, Yihang Huang, Jiayu Yang, Jianlan Ding, Chengxiang Jiang, Linxiao Ma

**Affiliations:** 1 School of Competitive Sports, Xi’an Physical Education University, Xi’an, China; 2 School of Physical Education and Training, Xi’an Physical Education University, Xi’an, China; 3 Beijing Xiannongtan Sports Technical School, Beijing, China; 4 Graduate School, Xi’an Physical Education University, Xi’an, China; 5 Key Laboratory of Sports Management and Cognitive Decision Making Brain Science of Shaanxi Universities, Xi'an, China; 6 School of Physical Education, Northeast Normal University, Changchun, China

**Keywords:** blood lactate monitoring, longitudinal case study, Olympic champion, periodization, race walking, training intensity distribution

## Abstract

This study examined the periodized training structure, training intensity distribution, key race-specific sessions, and blood lactate responses of a female Olympic champion in the 20 km race walk during preparation for the Paris 2024 Olympic Games. A longitudinal observational case study used training and physiological monitoring data collected from 4 December 2023 to 28 July 2024. The preparation period comprised winter preparation, competition-specific intensification, and final pre-competition phases. Race-walking mileage was classified into four practical intensity zones and grouped as low- (LIT), moderate- (MIT), and high-intensity training (HIT). Incremental road-based tests were used to establish lactate–speed relationships, with speeds at fixed blood lactate concentrations estimated by linear interpolation. Across the preparation period, the athlete completed 3,719.2 km of race-walking training, including 81.9% LIT, 9.4% MIT, and 8.7% HIT, indicating an overall pyramidal distribution. LIT remained dominant across all phases, whereas race-pace exposure increased during competition-specific intensification. Key sessions included 25–30 km long-distance race walking, repeated 1–2 km intervals, same-day double sessions, long-distance variable-pace training, and stair climbing combined with fast walking. From the first to the final monitoring session, estimated speed increased from 12.59 to 14.34 km h^−1^ at 2.0 mmol L^−1^ and from 13.68 to 15.79 km h^−1^ at 4.0 mmol L^−1^, while the slope between these reference points decreased from 1.85 to 1.38 mmol L^−1^ (km h^−1^)^−1^. These findings describe how individualized periodization, predominantly low-intensity training, race-specific sessions, lactate monitoring, environmental preparation, and competition strategy were integrated in Olympic preparation.

## 1 Introduction

Race walking is a technically constrained endurance event in which performance depends on the integrated development of aerobic capacity, movement economy, pacing control, and technical stability under fatigue. According to the current competition rules, athletes must maintain visible contact with the ground, and the advancing leg must be straightened from first contact until the vertical upright position (1). These technical constraints distinguish race walking from running and make performance dependent not only on physiological endurance, but also on the ability to sustain high walking velocity while maintaining legal and efficient technique. Previous biomechanical studies have shown that race-walking performance is closely related to stride length, stride frequency, joint motion, and coordination between the upper and lower body (2–5). Physiological evidence also indicates that lactate-threshold-related variables are associated with race-walking performance (6). Therefore, training for elite 20 km race walking requires the coordinated development of endurance capacity, race-specific speed, movement economy, and technical stability under fatigue.

Periodization provides a central framework for organizing training load, recovery, and competition-specific preparation in high-performance sport. In endurance events, the sequential arrangement of training phases allows coaches to develop general aerobic capacity, progressively introduce event-specific intensity, and regulate fatigue before major competitions (7, 9). Training intensity distribution is particularly important in this process because the relative proportion of low-, moderate-, and high-intensity training influences both physiological adaptation and fatigue accumulation (9, 10). Contemporary athlete-monitoring literature further emphasizes that external and internal load indicators should be integrated to support training decisions in high-performance settings (11). For elite race walkers, this issue is especially relevant because training must integrate high-volume aerobic work, threshold-oriented sessions, race-pace exposure, technical control under fatigue, and phase-specific load adjustment.

Blood lactate monitoring is commonly used in endurance training because it provides a practical indicator of the internal physiological response to a given external workload. In race walking, training intensity is usually prescribed and evaluated through pace, race-walking speed, or race-specific session design. However, speed alone does not fully describe the internal metabolic stress imposed on the athlete. Blood lactate concentration can therefore help coaches determine whether a given race-walking speed corresponds to low, moderate, or high physiological demand. When interpreted together with race-walking speed, training context, and phase-specific preparation goals, lactate data can support intensity-zone classification, pace prescription, and training-load regulation (12–15).

The lactate–speed relationship is particularly useful for longitudinal monitoring. If an athlete can sustain a higher race-walking speed at the same blood lactate concentration after a period of training, the lactate–speed relationship can be described as shifting rightward. Similarly, if blood lactate concentration increases more gradually as walking speed rises, the relationship can be described as having a flatter profile. In applied endurance settings, fixed lactate reference points, such as approximately 2 mmol L^−1^ and 4 mmol L^−1^, are often used to compare changes in speed across monitoring time points and to guide training intensity prescription (12, 13, 15). However, blood lactate concentration should not be interpreted as a simple or isolated marker of adaptation. Lactate responses are influenced by multiple biological and methodological factors, including exercise mode, sampling procedure, prior training load, nutritional status, environmental conditions, and individual athlete characteristics (16). Accordingly, lactate-derived indicators are most meaningful when they are interpreted within a consistent testing context and combined with training records, pace data, and the athlete's preparation goals.

Although periodization, training intensity distribution, and lactate-based monitoring have been widely discussed in endurance sports, longitudinal evidence from elite race walking remains limited. Existing studies have provided important insights into race-walking biomechanics, physiological characteristics, and competition performance, but fewer studies have documented how an Olympic-level 20 km race walker organizes training volume, intensity distribution, race-specific sessions, and physiological monitoring across an entire Olympic preparation cycle. This gap is practically important because elite race-walking preparation is not determined by a single training variable. Instead, performance emerges from the coordinated organization of phase-specific objectives, weekly and monthly training load, race-pace exposure, technical stability, environmental preparation, and individualized monitoring.

Therefore, the present study aimed to examine the periodized training structure, training intensity distribution, key race-specific training sessions, and blood lactate responses of a female Olympic champion 20 km race walker during preparation for the Paris 2024 Olympic Games. Specifically, this longitudinal case study sought to: (1) describe the phase division and training objectives across the Olympic preparation period; (2) quantify the distribution of race-walking mileage across intensity zones; (3) identify the functional role of key race-specific training sessions; and (4) analyze longitudinal changes in the lactate–speed relationship using fixed lactate reference points and road-based monitoring data. By integrating training records with blood lactate monitoring, this study provides applied evidence for understanding how individualized periodization and physiological load regulation can be organized in preparation for elite 20 km race walking.

## 2 Materials and methods

### 2.1 Study design

This study adopted a longitudinal observational case study design to examine the periodized training structure, training intensity distribution, key race-specific training sessions, and blood lactate responses of a female Olympic champion 20 km race walker during preparation for the Paris 2024 Olympic Games (17). The study was based on real-world training data collected throughout the athlete's pre-Olympic preparation period. Rather than using an experimentally controlled intervention, the present study analyzed the athlete's actual training process, with particular attention to phase-specific training goals, training load regulation, race-specific training content, physiological monitoring, and competition preparation.

### 2.2 Athlete profile

Jiayu Yang is an elite Chinese female 20 km race walker and the Paris 2024 Olympic champion. She has competed at the highest international level for several years, and her major competition results and awards over the past five years are summarized in Table 1. These results were used to contextualize the athlete's competitive level and to justify the relevance of this Olympic preparation case.

**Table 1:** Major competition results and awards of Jiayu Yang over the past five years.

Date	Location	Competition	Performance	Rank
20 Mar 2021	Huangshan, Anhui	National Race Walking Championships (Tokyo Olympic Trials), women's 20 km race walk; world record	1:23:49	1
24 Sep 2021	Xi’an, Shaanxi	14th National Games, women's 20 km race walk	1:27:14	1
29 Sep 2023	Hangzhou, Zhejiang	19th Asian Games, women's 20 km race walk	1:30:03	1
1 Aug 2024	Paris, France	Paris 2024 Olympic Games, women's 20 km race walk	1:25:54	1

Performance is expressed as hours:minutes:seconds. Rank indicates the athlete's final placing in the corresponding event. The listed results represent selected major competition achievements used to contextualize the athlete's elite competitive level during the observation period. The 20 March 2021 result was a world record in the women's 20 km race walk at the time of the competition. Competition results were obtained from official competition records and publicly available World Athletics records.

The case athlete, Jiayu Yang, is also a co-author of this manuscript. Her role in the study was primarily to provide access to training records, competition-related information, and contextual details regarding the preparation process, and to verify factual information related to her training and competition experience. Data organization, analysis, interpretation, and manuscript drafting were conducted by the other authors. This dual role is explicitly disclosed to ensure transparency in this participant-author case study.

### 2.3 Training period and phase division

The monitored preparation period extended from 4 December 2023 to 28 July 2024. According to the annual training plan, competition schedule, and phase-specific preparation goals, the training process was divided into three functional phases: winter preparation, competition-specific intensification, and final pre-competition phase. The detailed duration, objectives, and main training tasks of each phase are reported in the Results section.

### 2.4 Training data collection

Training data were collected through continuous follow-up by members of the national race-walking integrated support team. The recorded information included training plans, training load, race-walking mileage, training sessions, physiological indicators, competition performance, and coach feedback. Data were summarized weekly and then reorganized according to the three preparation phases to ensure continuity and consistency of the training record. This approach was consistent with athlete-monitoring recommendations emphasizing the combined interpretation of external load, internal response, recovery status, and coach-athlete feedback (11, 18–22).

Race-walking mileage was used as the main indicator of external training volume. Training sessions were categorized according to their training purpose, distance, pace, intensity characteristics, and role within the preparation period. Key race-specific sessions included 25–30 km long-distance race-walking sessions, 1–2 km repeated interval sessions, same-day double-session training, long-distance variable-pace sessions, altitude-based hill training, and stair climbing combined with fast walking. These sessions were analyzed to describe how training content was organized across phases and how race-specific capacity was progressively developed during the Olympic preparation period.

### 2.5 Training intensity classification and training intensity distribution

Training intensity was classified into four race-walking-specific zones according to the athlete's routine lactate–speed monitoring data and the pace ranges used in daily race-walking training: basic aerobic, aerobic, threshold-oriented, and race-pace intensity. For each preparation phase, race-walking mileage completed in each intensity zone was calculated and expressed as a percentage of the total race-walking mileage for that phase.

To facilitate comparison with the endurance-training literature, the four race-walking-specific zones were further grouped into a three-domain framework. Basic aerobic and aerobic training were classified as low-intensity training (LIT), threshold-oriented training was classified as moderate-intensity training (MIT), and race-pace intensity was classified as high-intensity training (HIT).

Training intensity distribution was then characterized descriptively according to the relative proportion of training volume completed in the three domains. A pyramidal TID was defined as a distribution in which LIT represented the largest proportion of training volume, followed by MIT and then HIT. A polarized TID was defined as a distribution with high proportions of LIT and HIT but relatively limited MIT, whereas a threshold-oriented TID was characterized by a comparatively greater contribution of MIT. This framework was used to describe the overall structure and phase-specific variation of the athlete's training program rather than to evaluate the superiority of any individual TID model (10, 23).

### 2.6 Blood lactate monitoring and analysis

Blood lactate (BLa) concentration was monitored as part of the athlete's routine physiological assessment during the Paris 2024 Olympic preparation period. Blood lactate responses provide practical information regarding metabolic demands under different exercise intensities and can assist in the interpretation of endurance performance when combined with external load indicators, such as walking speed and training volume (14).

In this study, blood lactate monitoring was integrated into the athlete's training monitoring system to characterize the relationship between race-walking speed and internal physiological response. The obtained lactate data were used to establish lactate–speed relationships, support training intensity classification, and evaluate longitudinal changes in metabolic responses across different stages of Olympic preparation.

#### 2.6.1 Lactate testing protocol

Incremental road-based race-walking tests were conducted on 27 December 2023, 18 January 2024, 30 March 2024, 15 May 2024, and 14 July 2024 as part of routine physiological monitoring during the athlete's preparation for the Paris 2024 Olympic Games. All tests were performed between 09:00 and 11:00 on outdoor roads used for routine race-walking training. During the 1–2 days preceding each test, the athlete completed relatively low-intensity training in accordance with the planned training schedule and maintained her usual dietary practices. Before testing, the athlete completed a 4-km race-walking warm-up on 27 December 2023, a 3-km warm-up on 18 January 2024, and a 5-km warm-up on 30 March, 15 May, and 14 July 2024.

Capillary blood samples were collected from the earlobe immediately after each incremental stage. Blood lactate concentration was measured using a Lactate Scout + portable lactate analyzer (SensLab GmbH, Leipzig, Germany; an EKF Diagnostics company) and expressed as mmol L^−1^. A test-specific interstage recovery interval was provided for blood sampling and data recording. The interstage interval varied according to the routine monitoring protocol used on each testing date. Stage distance and completion time were recorded, and race-walking speed was calculated from the actual completed distance and time. For the test conducted on 15 May 2024, race-walking speed values were obtained directly from the original physiological monitoring record because stage completion times were not retained.

Each test used progressively increasing race-walking speeds. Target pace was increased at successive stages according to the date-specific monitoring plan, while the athlete's actual completion time and calculated speed were used for subsequent analysis. The stage configuration varied across monitoring dates. The test conducted on 27 December 2023 comprised six 1,200-m stages followed by one 800-m stage, whereas the test conducted on 18 January 2024 comprised six 1,200-m stages. The test conducted on 30 March 2024 comprised five 2,000-m stages followed by one 1,200-m stage; the test conducted on 15 May 2024 comprised five 2,000-m stages; and the test conducted on 14 July 2024 comprised five 2,000-m stages followed by one 1,200-m stage. The stage-specific distances, race-walking speeds, and blood lactate concentrations are reported in Table 2.

**Table 2:** Stage-specific race-walking speeds and blood lactate responses during incremental road-based tests.

Date	Variable	Stage 1	Stage 2	Stage 3	Stage 4	Stage 5	Stage 6	Stage 7
27 Dec 2023	Distance (m)	1,200	1,200	1,200	1,200	1,200	1,200	800
	Speed (km h^−1^)	11.84	12.10	12.59	13.01	13.54	14.12	15.00
	BLa (mmol L^−1^)	1.3	1.7	2.0	2.6	3.7	5.0	6.7
18 Jan 2024	Distance (m)	1,200	1,200	1,200	1,200	1,200	1,200	—
	Speed (km h^−1^)	11.77	12.17	12.78	13.42	14.07	14.95	—
	BLa (mmol L^−1^)	1.3	1.4	1.7	2.7	3.3	5.9	—
30 Mar 2024	Distance (m)	2,000	2,000	2,000	2,000	2,000	1,200	—
	Speed (km h^−1^)	12.12	12.79	13.51	13.95	14.52	15.32	—
	BLa (mmol L^−1^)	0.9	1.2	1.9	2.3	3.2	4.4	—
15 May 2024	Distance (m)	2,000	2,000	2,000	2,000	2,000	—	—
	Speed (km h^−1^)	12.58	12.81	13.54	14.17	14.46	—	—
	BLa (mmol L^−1^)	1	1.2	1.9	2.7	3.3	—	—
14 Jul 2024	Distance (m)	2,000	2,000	2,000	2,000	2,000	1,200	—
	Speed (km h^−1^)	12.31	12.65	13.19	13.77	14.34	15.94	—
	BLa (mmol L^−1^)	1.2	1.3	1.3	1.7	2	4.2	—

BLa, blood lactate concentration. Blood samples were collected from the earlobe immediately after each incremental stage. Race-walking speed was calculated from the recorded stage distance and completion time, except for the test conducted on 15 May 2024, for which speed values were obtained directly from the original monitoring record. The protocols varied slightly across testing dates as part of routine road-based physiological monitoring. An em dash indicates that the stage was not performed.

Because the tests were embedded in routine road-based physiological monitoring and the stage configurations and interstage recovery intervals were not identical across all testing dates, the resulting lactate–speed relationships were used for descriptive longitudinal comparison, training-intensity classification, and pace prescription rather than for the determination of individualized physiological thresholds such as LT1 or LT2.

#### 2.6.2 Lactate data processing and interpretation

The blood lactate values obtained from the incremental road-based race-walking tests were matched with the corresponding race-walking speeds calculated from the actual completed stage distance and time. These paired data were used to establish an individual lactate–speed relationship at each monitoring time point. The position and slope of the lactate–speed relationships were then compared descriptively to evaluate longitudinal changes in the athlete's internal physiological response to increasing race-walking speed during the preparation period.

Fixed blood lactate concentrations of 2.0 and 4.0 mmol L^−1^ were used as operational reference points for training-intensity classification and longitudinal comparison. The fixed 2.0 mmol L^−1^ reference point was used as the practical boundary between low-intensity and threshold-oriented training, whereas the fixed 4.0 mmol L^−1^ reference point was used as the practical boundary between threshold-oriented and race-pace intensity. These reference points were used to support the interpretation of training intensity zones and pace prescription and were not considered individually determined physiological thresholds such as LT1 or LT2 (12, 13).

Based on the lactate–speed relationship and the pace ranges used in routine training, four race-walking intensity zones were established: basic aerobic, aerobic, threshold-oriented, and race-pace intensity. These zones were subsequently integrated with the three-domain training intensity distribution framework described in Section 2.5. Specifically, basic aerobic and aerobic training were classified as low-intensity training (LIT), threshold-oriented training as moderate-intensity training (MIT), and race-pace intensity as high-intensity training (HIT).

For longitudinal comparison, the race-walking speeds corresponding to fixed blood lactate concentrations of 2.0, 3.0, and 4.0 mmol L^−1^ were estimated by linear interpolation between adjacent measured stages. The fixed concentration of 3.0 mmol L^−1^ was included as an intermediate reference point to describe changes within the moderate blood lactate range. No extrapolation beyond the highest or lowest measured blood lactate concentration was performed. Accordingly, the speed at 4.0 mmol L^−1^ was not estimated for the test conducted on 15 May 2024 because the highest measured blood lactate concentration during that session was 3.3 mmol L^−1^. The resulting indicators were reported as the estimated speeds at 2.0, 3.0, and 4.0 mmol L^−1^, respectively.

Absolute and percentage changes in the estimated speeds at 2.0 and 4.0 mmol L^−1^ were calculated relative to the first monitoring time point. The speed difference between the two reference points was calculated as the speed at 4.0 mmol L^−1^ minus the speed at 2.0 mmol L^−1^. The slope between 2.0 and 4.0 mmol L^−1^ was calculated as 2.0 divided by the difference between the estimated speeds at 4.0 and 2.0 mmol L^−1^ and was expressed as mmol L^−1^ (km h^−1^)^−1^. A higher estimated speed at a fixed blood lactate concentration was interpreted descriptively as a rightward shift of the lactate–speed relationship, whereas a lower slope between 2.0 and 4.0 mmol L^−1^ was interpreted as a flatter lactate response profile. Because the tests were conducted on outdoor roads as part of routine physiological monitoring and differed in stage configuration and interstage recovery duration, these indicators were used only for descriptive longitudinal comparison and should not be interpreted as laboratory-derived physiological threshold parameters.

## 3 Results

### 3.1 Phase division and training objectives

The Paris 2024 Olympic preparation period was organized into three functional phases according to the annual training plan, competition schedule, and phase-specific preparation goals: the winter preparation phase, the competition-specific intensification phase, and the final pre-competition phase. The duration, training objectives, and main training tasks of each phase are summarized in Table 3. This phase-based organization was consistent with contemporary periodization and training-planning principles for high-performance endurance sport (7–10).

**Table 3:** Phase division, training objectives, and main training tasks during Paris 2024 Olympic preparation.

Phase	Duration	Training objectives	Main training tasks
Winter preparation phase	4 Dec 2023–10 Mar 2024; 14 weeks	1. Develop aerobic capacity and improve 30 km long-distance tolerance. 2. Refine race-walking technique, especially hip rotation and landing mechanics. 3. Improve basic strength and proprioceptive control.	1. 25–30 km long-distance aerobic race-walking sessions. 2. Strength training and proprioceptive training. 3. Long-distance aerobic walking combined with short-distance technical work. 4. Low-intensity variable-pace walking.
Competition-specific intensification phase	11 Mar 2024–26 May 2024; 11 weeks	1. Improve race-specific aerobic–anaerobic capacity and adapt to a steady-pace-plus-acceleration race pattern. 2. Enhance late-race lactate tolerance. 3. Evaluate training effects through competition.	1. Variable-pace long-distance training. 2. 1.5–2 km repeated interval sessions. 3. Blood lactate monitoring. 4. Split training sessions.
Final pre-competition phase	27 May 2024–28 Jul 2024; 9 weeks	1. Regulate competitive readiness and adapt to hot and humid conditions expected in Paris. 2. Improve lactate tolerance and sustained walking capacity under altitude conditions. 3. Reduce accumulated fatigue before competition.	1. Short-distance hill walking. 2. Altitude-based training. 3. Same-day double-session training. 4. Progressive acceleration walking before competition.

Phase division was based on the athlete's Olympic preparation plan, competition schedule, and phase-specific training objectives. Duration is expressed in calendar weeks. Training objectives and main training tasks were summarized from the athlete's training records and coaching plan.

The winter preparation phase lasted from 4 December 2023 to 10 March 2024. This phase was primarily designed to build a broad aerobic base, improve long-distance tolerance, and address technical limitations. Training during this phase was dominated by relatively high volume and low-to-moderate intensity, providing a foundation for subsequent increases in race-pace intensity.

The competition-specific intensification phase lasted from 11 March to 26 May 2024. Building on the aerobic foundation developed during winter preparation, this phase aimed to improve race-specific aerobic–anaerobic capacity, adapt the athlete to a steady-pace-plus-acceleration race pattern, and enhance late-race lactate tolerance. Training content included long-distance variable-pace sessions, repeated interval sessions, split sessions, and lactate-based monitoring.

The final pre-competition phase lasted from 27 May to 28 July 2024 and represented the final stage of Olympic preparation. This phase focused on maintaining race-specific capacity, consolidating competition rhythm, and regulating competition readiness before the Olympic race. According to the training arrangement and environmental conditions, this phase consisted of three sequential periods: pre-altitude preparation (27 May–10 June), moderate-altitude training (11 June–11 July), and heat acclimation with final competition adjustment (12 July–28 July).

During the pre-altitude preparation period, training continued the race-specific structure established in the previous phase, with appropriate training volume and specialized stimuli maintained before entering the altitude environment. From 11 June to 11 July 2024, Jiayu Yang completed a moderate-altitude training period in Roccaraso, Italy. During this period, the athlete continued race-walking-specific training, including long-distance sessions, interval training, variable-pace sessions, and race-pace training, while training load and session organization were adjusted according to the characteristics of the preparation phase. From 12 July to 28 July 2024, the athlete entered the heat acclimation and final competition adjustment period in Lido di Ostia, Rome, Italy. Training during this period was conducted under hot and humid environmental conditions, with cooling strategies and core temperature monitoring incorporated into the training process. Compared with the previous phases, the training objective gradually shifted from further development of physical capacity toward the maintenance of race-specific performance and optimization of competition readiness.

### 3.2 Competition context and planned pacing strategy

**Figure 1:**
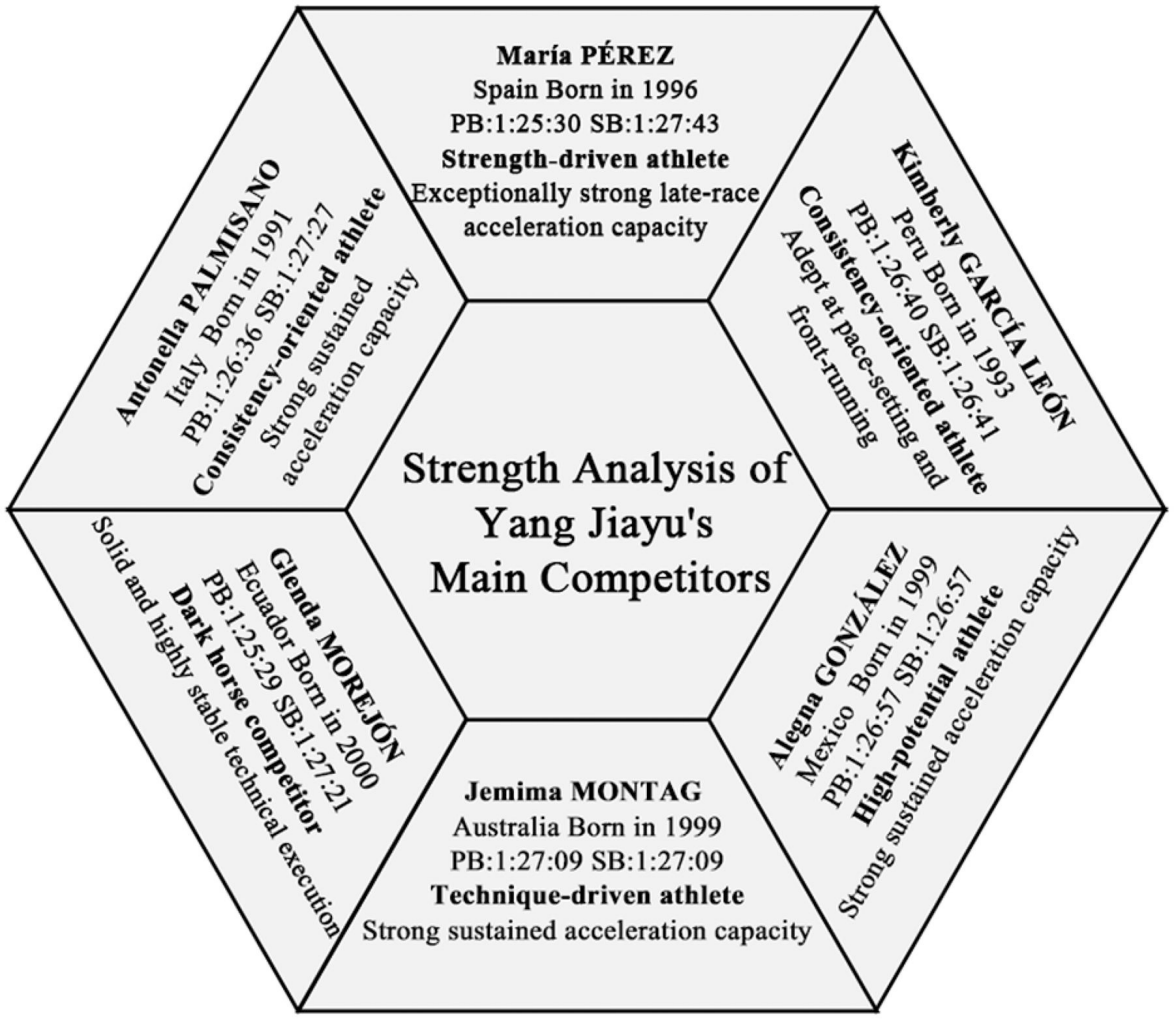
Competitive profile of main rivals in the Paris 2024 Olympic women's 20 km race walk. The figure summarizes the pre-competition assessment of the principal competitors based on recent performance level, personal-best performance, competitive consistency, technical characteristics, pacing tendencies, and potential tactical threat. Performance information was derived from official competition records available before the Paris 2024 Olympic Games, whereas technical and tactical characteristics were qualitatively assessed by the coaching and integrated support team based on recent race performances and competition-video analysis. This assessment was used to contextualize the planned pacing strategy and performance target.

Before the Paris 2024 Olympic women's 20 km race walk, the main rivals were analyzed in relation to their recent performance level, competitive stability, and potential threat during the race. This pre-competition competitive profile is summarized in Figure 1. The analysis helped inform the planned race strategy, particularly the need to avoid passive following, control the early race rhythm, and prepare for a decisive acceleration in the middle or later stages.

The pre-competition target was approximately 1:26:00. The planned pacing strategy emphasized a controlled opening phase, progressive speed increase, and a strong final 5 km. The planned 5-km split times were 22:05, 21:40, 21:15, and 21:05, with a planned split total of 1:26:05.

The actual Olympic performance was 1:25:54, which was 11 s faster than the planned split total. As shown in Table 4, the athlete completed the first 5 km in 21:34, 31 s faster than planned, while remaining with the leading group. The 5–10 km section was also faster than planned, reflecting an early breakaway and the establishment of a clear lead. The 10–15 km section was slower than planned, indicating a controlled middle phase under heat, hydration, and race-management demands. The final 5 km was completed in 21:07, almost identical to the planned 21:05, showing effective late-race speed maintenance despite the chase from competitors.

**Table 4:** Planned and actual 5-km split pacing in the Paris 2024 Olympic women's 20 km race walk.

Race section	Planned split time	Planned pace (min km^−1^)	Actual split time	Actual pace (min km^−1^)	Difference (actual—planned)	Tactical interpretation
0–5 km	22:05	4:25	21:34	4:19	−0:31	Faster-than-planned opening while remaining with the leading group
5–10 km	21:40	4:20	21:32	4:18	−0:08	Early breakaway and establishment of a clear lead
10–15 km	21:15	4:15	21:41	4:20	+0:26	Controlled middle phase under heat, hydration, and race-management demands
15–20 km	21:05	4:13	21:07	4:13	+0:02	Strong final phase while maintaining speed under pressure from pursuing competitors
Overall	1:26:05	4:18	1:25:54	4:18	−0:11	Flexible execution characterized by an early breakaway, mid-race control, and effective late-race speed maintenance

Split time is expressed as minutes:seconds, except for the overall performance, which is expressed as hours:minutes:seconds. Pace values were calculated from the corresponding 5-km split times and rounded to the nearest second per kilometer. Difference was calculated as actual split time minus planned split time; negative values indicate that the actual split was faster than planned, whereas positive values indicate that it was slower than planned.

Overall, the actual pacing pattern differed from the original progressive plan but achieved the intended performance target. It reflected a flexible race strategy characterized by early breakaway, mid-race control, and strong final execution, consistent with the importance of stage-specific pacing and late-race speed maintenance in elite race walking (24).

### 3.3 Training intensity distribution across preparation phases

Training intensity was classified into four race-walking intensity zones: basic aerobic, aerobic, threshold-oriented, and race-pace intensity. The approximate blood lactate ranges, race-walking speeds, pace ranges, training functions, and corresponding three-domain classifications for these zones are summarized in Table 5. Basic aerobic and aerobic training were grouped as low-intensity training (LIT), threshold-oriented training as moderate-intensity training (MIT), and race-pace intensity as high-intensity training (HIT).

**Table 5:** Practical lactate reference points, race-walking speed and pace ranges, and training intensity classification.

Training zone	Blood lactate reference (mmol L^−1^)	Speed range (km h^−1^)	Equivalent pace range (min km^−1^)	Relation to practical lactate reference points	Three-domain classification	Main training function
Basic aerobic	∼1.2	11.4–11.8	5:15–5:05	Below the first operational lactate reference point	LIT	Low-intensity volume accumulation and aerobic activation
Aerobic	∼2.0	11.8–12.9	5:05–4:40	Around the first operational lactate reference point	LIT	Aerobic endurance development and sTABLE-pace training
Threshold-oriented	3.0–3.5	12.9–13.7	4:40–4:25	Between the first operational lactate reference point and the operational OBLA reference point	MIT	Threshold-related training and development of sustained race-walking capacity
Race-pace intensity	4.0–5.0	13.8–14.7	4:20–4:05	At or above the operational OBLA reference point	HIT	Race-pace exposure and high-intensity race-specific stimulation

Blood lactate values are approximate operational reference points used to classify training intensity and guide race-walking pace prescription during routine road-based monitoring. The first operational lactate reference point was set at approximately 2.0 mmol L^−1^, while the operational OBLA reference point was set at approximately 4.0 mmol L^−1^. These values were used for practical training regulation and should not be interpreted as laboratory-derived threshold parameters. Speed and pace ranges were derived from the athlete's training records and coaching prescription. LIT, low-intensity training; MIT, moderate-intensity training; HIT, high-intensity training; OBLA, onset of blood lactate accumulation.

**Figure 2:**
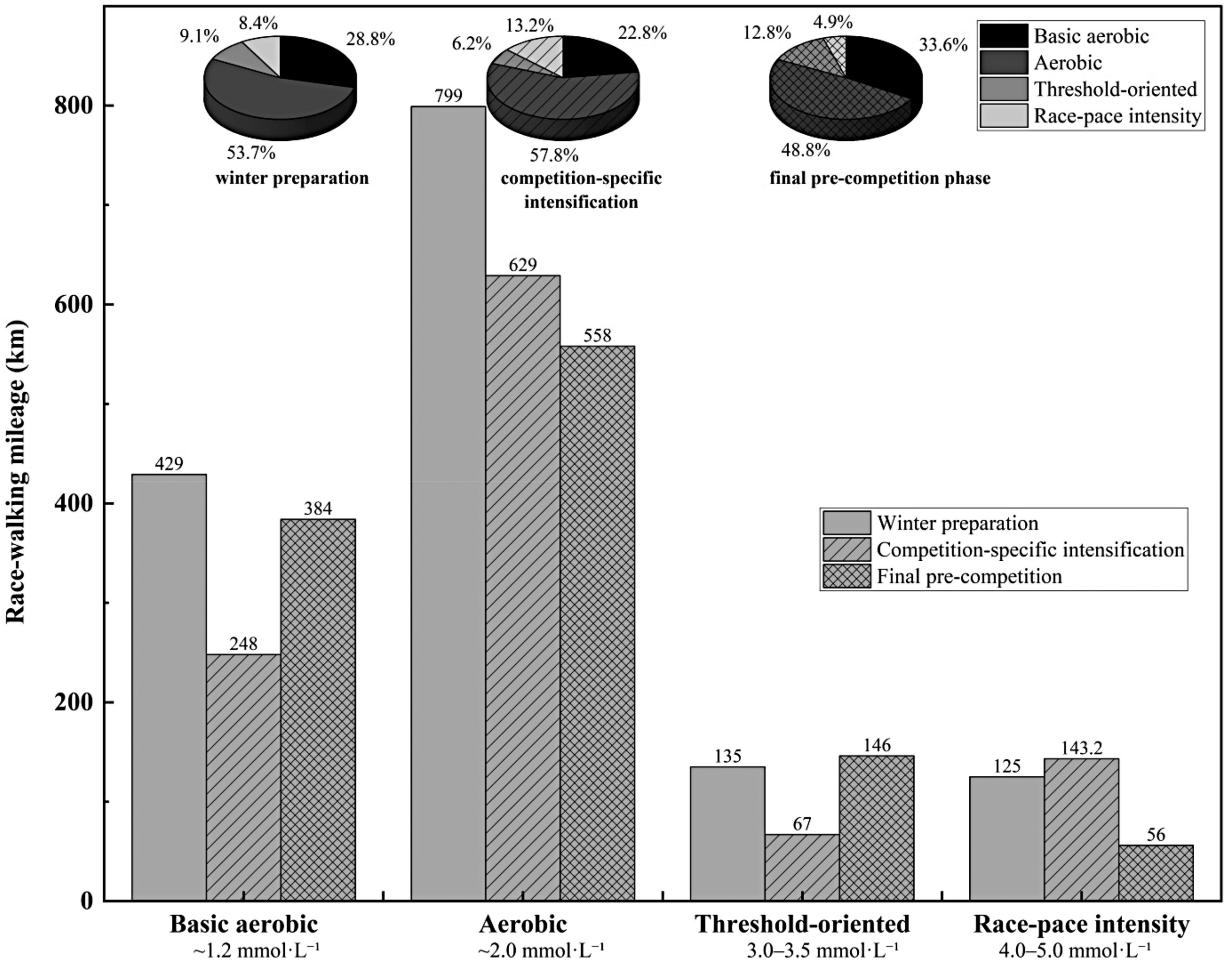
Race-walking training intensity distribution across the three preparation phases. The grouped bar chart presents race-walking mileage completed in the basic aerobic, aerobic, threshold-oriented, and race-pace intensity zones during the winter preparation, competition-specific intensification, and final pre-competition phases. The pie charts show the percentage distribution of the four intensity zones within each preparation phase. Basic aerobic and aerobic training were grouped as low-intensity training (LIT), threshold-oriented training as moderate-intensity training (MIT), and race-pace intensity as high-intensity training (HIT). Blood lactate values represent approximate operational reference concentrations and practical ranges used for training-intensity classification rather than individually determined physiological thresholds. Values above the bars indicate race-walking mileage in kilometers. Percentages may not sum to exactly 100% because of rounding.

Across the Paris 2024 Olympic preparation period, the athlete completed a total of 3,719.2 km of race-walking training. LIT accounted for 3,047.0 km, representing 81.9% of total race-walking mileage. MIT accounted for 348.0 km, or 9.4%, whereas HIT accounted for 324.2 km, or 8.7%. The overall training intensity distribution was therefore classified as pyramidal, with LIT representing the largest proportion of race-walking mileage, followed by MIT and HIT. The phase-specific mileage and proportional distribution across the four race-walking intensity zones are presented in Figure 2.

During the winter preparation phase, total race-walking mileage reached 1,488.0 km. Basic aerobic training accounted for 429.0 km, or 28.8% of the phase total, whereas aerobic training accounted for 799.0 km, or 53.7%. Together, the two low-intensity zones accounted for 1,228.0 km, representing 82.5% of total phase mileage. Threshold-oriented training accounted for 135.0 km, or 9.1%, and race-pace intensity accounted for 125.0 km, or 8.4%. This phase displayed a clear pyramidal distribution.

During the competition-specific intensification phase, total race-walking mileage was 1,087.2 km. Basic aerobic training accounted for 248.0 km, or 22.8%, and aerobic training accounted for 629.0 km, or 57.8%. Together, these two low-intensity zones accounted for 877.0 km, representing 80.6% of total phase mileage. Threshold-oriented training accounted for 67.0 km, or 6.2%, whereas race-pace intensity accounted for 143.2 km, or 13.2%. Compared with the winter preparation phase, the proportion of race-pace intensity increased, while LIT remained above 80% of total race-walking mileage. Although this phase did not follow the strict pyramidal ordering of LIT > MIT > HIT, the distribution remained strongly dominated by LIT and reflected a phase-specific increase in race-pace exposure.

During the final pre-competition phase, total race-walking mileage was 1,144.0 km. Basic aerobic training accounted for 384.0 km, or 33.6%, and aerobic training accounted for 558.0 km, or 48.8%. Together, the two low-intensity zones accounted for 942.0 km, representing 82.4% of total phase mileage. Threshold-oriented training accounted for 146.0 km, or 12.8%, whereas race-pace intensity accounted for 56.0 km, or 4.9%. This phase again displayed a pyramidal distribution, with LIT accounting for the largest proportion of total race-walking mileage.

As illustrated in Figure 2, LIT remained dominant throughout all three preparation phases. However, the relative contribution of MIT and HIT varied according to the objectives of each phase. Race-pace exposure was greatest during the competition-specific intensification phase, whereas threshold-oriented mileage was highest during the final pre-competition phase.

### 3.4 Monthly race-walking mileage during Olympic preparation

To further describe the external training load during the Olympic preparation period, monthly race-walking mileage from December 2023 to July 2024 was compared with that recorded during the preceding 2023 training year, which extended from December 2022 to October 2023. The 2023 training year was used as an individual historical reference for the athlete's non-Olympic preparation pattern, whereas the December 2023–July 2024 data represented the Paris 2024 Olympic preparation period.

**Figure 3:**
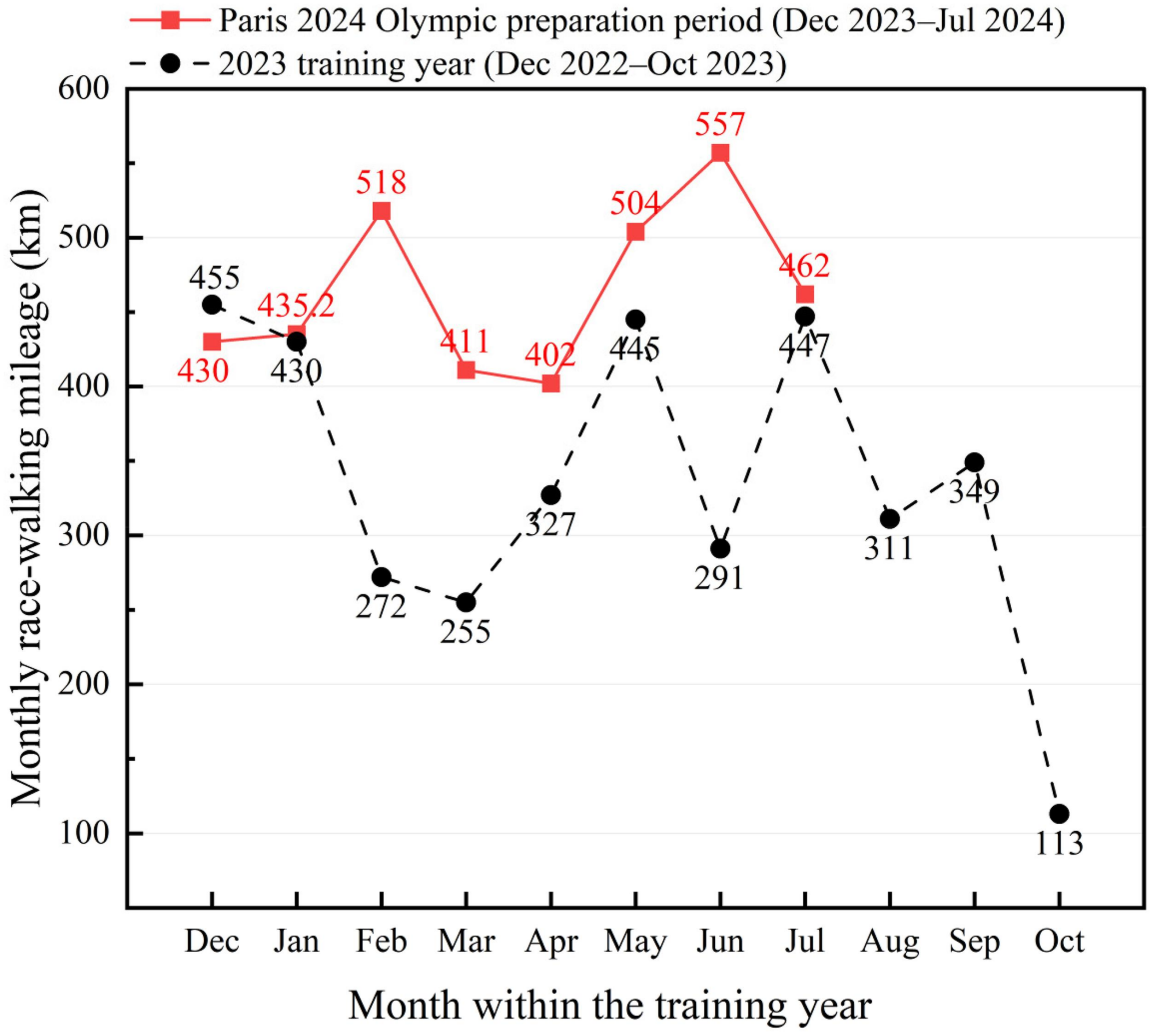
Monthly race-walking mileage during the Paris 2024 Olympic preparation period and the 2023 training year. The solid line represents monthly race-walking mileage during the Paris 2024 Olympic preparation period from December 2023 to July 2024. The dashed line represents monthly race-walking mileage during the preceding 2023 training year from December 2022 to October 2023. The two series are aligned according to month within their respective training years to illustrate differences in monthly training volume and its distribution. Values are expressed in kilometers.

As shown in Figure 3, monthly race-walking mileage during the 2023 training year fluctuated substantially, ranging from 113 to 455 km and displaying an alternating high–low pattern. In contrast, monthly mileage during the Paris 2024 Olympic preparation period ranged from 402 to 557 km and remained above 400 km in most months. This pattern indicated a higher baseline volume and a more stable accumulation of race-walking mileage during the Olympic preparation period.

The Paris 2024 Olympic preparation pattern therefore showed three main characteristics: a higher baseline race-walking volume, a more stable monthly distribution, and a sustained high-volume preparation structure. This organization provided the volume foundation for subsequent race-specific training and final pre-competition regulation.

### 3.5 Key race-specific training sessions and their functional roles

During the Paris 2024 Olympic preparation period, the coaching team conducted a systematic assessment of the athlete's race-specific capacity on the basis of previous international competition performances, competition videos, physiological and biochemical monitoring, and historical training load data. This assessment identified two key issues requiring targeted training adjustment: insufficient late-race acceleration capacity and a tendency toward loss of contact in some technical phases. Accordingly, pre-competition training emphasized the consolidation of aerobic capacity while improving late-race speed maintenance and technical stability.

The design of training methods was also informed by threshold-oriented endurance training principles. By regulating intensity and recovery rhythm, the training program aimed to allow the athlete to complete high-quality work within the practical intensity ranges used for threshold-oriented and race-pace training while using selected high-intensity stimuli to improve lactate tolerance and race-pace sustainability (12, 13, 25–28).

#### 3.5.1 Long-distance race-walking sessions

Long-distance sessions were used as a foundational method for developing aerobic capacity, race-specific endurance, muscular endurance, and stable pacing rhythm. Across the preparation period, the athlete completed 11 key long-distance sessions of 25–30 km, including three 30-km sessions. These sessions were more frequent and longer than in her previous training pattern. During winter preparation, long-distance sessions were conducted at relatively slower paces and were mainly used to build aerobic depth. Average pace ranged from 4:52 to 5:11 min km^−1^, corresponding approximately to a blood lactate concentration of 1.2–2.0 mmol L^−1^. The relatively stable pacing and the absence of large speed fluctuations indicated controlled load intensity. Split data also showed that in many sessions the later segments were stable or slightly faster than earlier segments, suggesting good fatigue resistance and pacing control during prolonged walking. Detailed session data are presented in Table 6.

**Table 6:** Key long-distance race-walking sessions and segment split times.

Distance	Date	Completion time	Average pace (min km^−1^)	0–5 km	5–10 km	10–15 km	15–20 km	20–25 km	Final segment
30 km	18 Dec 2023	2:35:34	5:11	25:50	25:45	25:40	25:35	25:30	25:20 (25–30 km)
30 km	1 Feb 2024	2:26:02	4:52	24:43	24:26	24:23	24:12	23:58	24:18 (25–30 km)
30 km	9 Jun 2024	2:32:43	5:05	25:41	25:42	25:38	25:25	25:24	25:21 (25–30 km)
28 km	6 May 2024	2:19:01	5:00	25:20	25:17	24:54	24:40	24:00	14:41 (25–28 km)
25 km	14 Dec 2023	2:09:34	5:10	25:56	26:00	26:02	25:49	25:47	—
25 km	21 Dec 2023	2:07:09	5:05	25:55	25:54	25:22	25:05	25:03	—
25 km	17 Feb 2024	2:02:35	4:54	25:28	24:19	24:18	24:16	24:13	—
25 km	3 May 2024	2:07:57	5:05	25:41	25:42	25:38	25:25	25:24	—
25 km	4 Jun 2024	2:07:55	5:07	—	—	—	—	—	—
25 km	16 Jun 2024	2:07:20	5:06	—	—	—	—	—	—
25 km	23 Jun 2024	2:03:27	4:56	—	—	—	—	—	—

Segment split times are reported according to the actual distance interval covered in each segment. For 25-km sessions, five 5-km split times are reported when available. For 30-km sessions, the final segment represents 25–30 km. For the 28-km session on 6 May 2024, the final segment represents 25–28 km. A dash indicates that segment split data were not available.

#### 3.5.2 Repeated interval sessions

Repeated interval sessions were performed over 1 km, 1.5 km, and 2 km distances, with recovery intervals ranging from 30 to 60 s. The 1.5-km sessions were conducted during the winter preparation phase (Table 7). On 16 December 2023, the athlete completed 10 repetitions with 60-s recovery, with repetition times ranging from 7:23 to 7:32. On 30 December 2023, six repetitions were completed with the same recovery interval, with repetition times ranging from 7:26 to 7:32.

**Table 7:** 1.5-km repeated rhythm sessions.

Date	Recovery	1	2	3	4	5	6	7	8	9	10
16 Dec 2023	60 s	7:23	7:23	7:27	7:32	7:27	7:27	7:26	7:25	7:27	7:24
30 Dec 2023	60 s	7:30	7:29	7:32	7:26	7:30	7:27	—	—	—	—

Values in columns 1–10 represent the completion time of each 1.5-km repetition. Recovery indicates the recovery interval between repetitions. A dash indicates that the corresponding repetition was not performed or was not recorded in the original training log.

The 2-km repeated sessions were mainly completed during the winter preparation and competition-specific intensification phases (Table 8). On 20 December 2023, eight 2-km repetitions were completed with 60-s recovery, with times ranging from 9:51 to 9:58. On 23 December 2023, 10 repetitions were recorded, with times ranging from 8:57 to 10:00. During the competition-specific intensification phase, the session on 30 March 2024 included five 2-km repetitions followed by a final 1,200-m repetition, with the 2-km repetition times ranging from 8:16 to 9:55 and the final 1,200-m repetition completed in 4:42.

**Table 8:** 2-km repeated rhythm sessions.

Date	Recovery	1	2	3	4	5	6	7	8	9	10
20 Dec 2023	60 s	9:56	9:58	9:55	9:51	9:54	9:54	9:54	9:57	—	—
23 Dec 2023	60 s	9:55	9:24	8:57	10:00	9:55	9:54	9:55	9:55	9:54	9:50
30 Mar 2024	60 s	9:55	9:24	8:54	8:36	8:16	4:42 (1,200 m)	—	—	—	—

Values in columns 1–10 represent the completion time of each 2-km repetition unless otherwise indicated. Recovery indicates the recovery interval between repetitions. For the session on 30 March 2024, the sixth repetition was 1,200 m rather than 2 km and is therefore reported as 4:42 (1,200 m). A dash indicates that the corresponding repetition was not performed or was not recorded in the original training log.

The 1-km repeated sessions were performed during both the winter preparation and final pre-competition phases (Table 9). During January 2024, 10 repetitions were completed at approximately 4:37–4:42 min km^−1^, with recovery intervals ranging from 45 to 60 s. In the final pre-competition phase, three 1-km sessions were completed on 18, 22, and 25 June 2024. Recovery intervals were progressively reduced from 60 s to 45 s and then 30 s. Across these sessions, most repetition times ranged from 4:24 to 4:34 min km^−1^, with the final repetition on 25 June completed in 4:10. Repeated interval sessions were used to improve race-specific speed, lactate tolerance, and the ability to maintain technique and velocity under fatigue. Such sessions are consistent with the broader role of interval and speed-endurance training as stimuli for physiological adaptation and performance improvement in trained athletes (25–27, 29). These sessions included 1-km, 1.5-km, and 2-km repeated rhythm work, with the recovery interval progressively shortened in selected pre-competition sessions.

**Table 9:** 1-km repeated rhythm session**s.**

Date	Recovery	1	2	3	4	5	6	7	8	9	10
5 Jan 2024	60 s	4:38	4:40	4:40	4:38	4:40	4:40	4:40	4:40	4:40	4:39
11 Jan 2024	45 s	4:42	4:37	4:38	4:39	4:41	4:40	4:39	4:39	4:39	4:37
27 Jan 2024	58 s	4:19	4:22	4:23	4:22	4:21	4:22	4:20	4:21	4:21	4:21
18 Jun 2024	60 s	4:33	4:32	4:28	4:31	4:27	4:30	4:28	4:30	4:28	4:27
22 Jun 2024	45 s	4:28	4:34	4:27	4:29	4:28	4:27	4:28	4:25	4:27	4:26
25 Jun 2024	30 s	4:28	4:27	4:28	4:28	4:27	4:28	4:26	4:28	4:24	4:10

Values in columns 1–10 represent the completion time of each 1-km repetition. Recovery indicates the recovery interval between repetitions. A dash indicates that the corresponding repetition was not performed or was not recorded in the original training log.

#### 3.5.3 Same-day double-session training

Same-day double-session training refers to arranging two or more race-walking sessions within the same day. In endurance training, concentrated or repeated daily sessions can be used to increase cumulative stimulus and challenge recovery capacity, although such strategies must be carefully controlled to avoid excessive fatigue (11, 20, 21, 30). In this preparation period, such sessions were mainly concentrated in the winter preparation and final pre-competition phases. Detailed session data are presented in Table 10.

**Table 10:** Same-day double-session trainin**g.**

Date	Distance	Time	Pace	Split 1	Split 2	Split 3	Split 4
2 Jan 2024	15 km (morning)	1:12:53	4:52	24:46	24:15	23:53	—
2 Jan 2024	15 km (afternoon)	1:12:50	4:51	24:46	24:04	23:59	—
6 Jan 2024	15 km (morning)	1:13:03	4:53	19:55 (4 km)	29:11 (6 km)	23:56	—
6 Jan 2024	10 km (afternoon)	48:12	4:50	24:21	23:50	—	—
30 Jan 2024	12 km (morning)	55:29	4:35	23:13	22:56	9:19	—
30 Jan 2024	12 km (afternoon)	55:36	4:36	23:06	23:13	9:16	—
20 Jun 2024	12 km (morning)	56:14	4:39	—	—	—	—
20 Jun 2024	12 km (afternoon)	56:03	4:38	—	—	—	—
9 Jan 2024	20 km (morning)	1:37:27	4:52	24:50	24:24	24:20	23:52
9 Jan 2024	5 km (afternoon)	25:38	—	—	—	—	—
16 Jan 2024	20 km (morning)	1:36:20	4:50	24:54	24:52	23:50	22:44
16 Jan 2024	5 km (afternoon)	24:55	—	—	—	—	—

Morning and afternoon indicate two race-walking sessions performed on the same day. Distance, time, and pace refer to each individual session rather than the combined daily total. Split 1–Split 4 represent consecutive segment split times recorded in the original training log. Unless otherwise indicated, split times are reported for 5-km segments; segment distances that differ from 5 km are shown in parentheses, such as 19:55 (4 km), 29:11 (6 km), or 9:19 (2 km). A dash indicates that the corresponding split was not recorded or was not applicable.

#### 3.5.4 Long-distance variable-pace sessions

Long-distance variable-pace sessions combined aerobic endurance development with race-specific speed stimulation. Compared with constant-pace long-distance walking, these sessions placed greater emphasis on speed changes, rhythm control, and late-session tolerance. They also provided a bridge between high-volume aerobic work and race-pace exposure, which is consistent with endurance training evidence emphasizing the interaction between volume, intensity, and session-specific goals (9, 10, 23, 25–28, 31, 32).

The variable-pace sessions were completed with generally high quality. In several sessions, the athlete maintained stable pace after faster segments, suggesting the ability to sustain stride rhythm and technical control despite fatigue accumulation. Some sessions also showed a stronger finishing pattern, which is consistent with the late-race acceleration tendency often observed in high-level race walking. Detailed session data are presented in Table 11.

**Table 11:** Long-distance variable-pace training sessions.

Date	Total distance	Time	Pace	Segment 1	Segment 2	Segment 3	Segment 4
7 Feb 2024	25 km	2:03:27	4:56	48:45 (10 km)	4:30 (1 km × 5)	48:52 (10 km)	—
2 Apr 2024	25 km	1:58:33	4:44	48:33 (10 km)	9:03 (2 km × 1)	37:39 (8 km)	23:18 (5 km × 1)
7 Apr 2024	25 km	2:01:40	4:52	38:54 (8 km)	4:24 (1 km × 5)	33:21 (7 km)	4:20 (1 km × 5)
10 May 2024	15 km	1:06:09	4:25	49:39 (10 km)	4:30 (1 km × 5)	—	—
27 Jun 2024	18 km	1:21:57	4:33	28:02 (6 km)	4:30–4:25 (1 km × 6)	27:21 (6 km)	—
19 Jul 2024	20 km	1:36:02	4:48	39:09 (8 km)	9:12 (2 km × 1)	38:43 (8 km)	9:05 (2 km × 1)

Segment 1–Segment 4 represent consecutive continuous or variable-pace components recorded in the original training log. Values are reported as time with the corresponding distance or repetition structure in parentheses. For continuous segments, the value indicates the completion time for that segment, such as 48:45 (10 km). For repeated short-distance variable-pace components, the value indicates the target or recorded time for each repetition, with the repetition distance and number shown in parentheses, such as 4:30 (1 km × 5) or 9:03 (2 km × 1). Total distance and total time refer to the complete training session. A dash indicates that the corresponding segment was not performed or was not recorded.

#### 3.5.5 Stair climbing combined with fast walking

Stair climbing combined with fast walking was used as a compound training method integrating lower-limb strength endurance, lactate-tolerance stimulation, and race-specific speed work. The session design generally involved repeated stair-climbing sets followed by fast walking, with blood lactate responses monitored during the session. This type of supplementary strength-endurance work was consistent with evidence that strength training can support endurance performance and movement economy when appropriately integrated with endurance training (33–35).

During the preparation period, the training stimulus was mainly created by approximately 450 stair steps per set. Blood lactate was monitored after each set, and subsequent training intensity was regulated according to the lactate response. After 8–10 sets of stair climbing, the athlete immediately completed 5–6 km of fast walking before full recovery. This design simulated late-race fatigue and aimed to improve late-race speed endurance, lower-limb muscular endurance, and higher race-walking speeds at comparable blood lactate concentrations

### 3.6 Activation, strength, and proprioceptive training

In addition to race-walking sessions, the preparation program included activation, strength, and proprioceptive training designed to support technical stability, movement economy, and injury prevention. This complementary work was consistent with the importance of movement economy and segmental coordination in endurance performance and race walking (25, 33–35).

Strength training focused on the lower limbs and trunk, reflecting the propulsion and postural-control demands of race walking. Training methods included body-weight loading, elastic resistance, and constant external loading. Lower-limb exercises targeted propulsion and landing control, whereas trunk exercises were used to reinforce pelvic and core stability during high-speed walking. This organization was intended to improve force transmission and reduce excessive compensatory movement during prolonged race-walking efforts.

**Figure 4:**
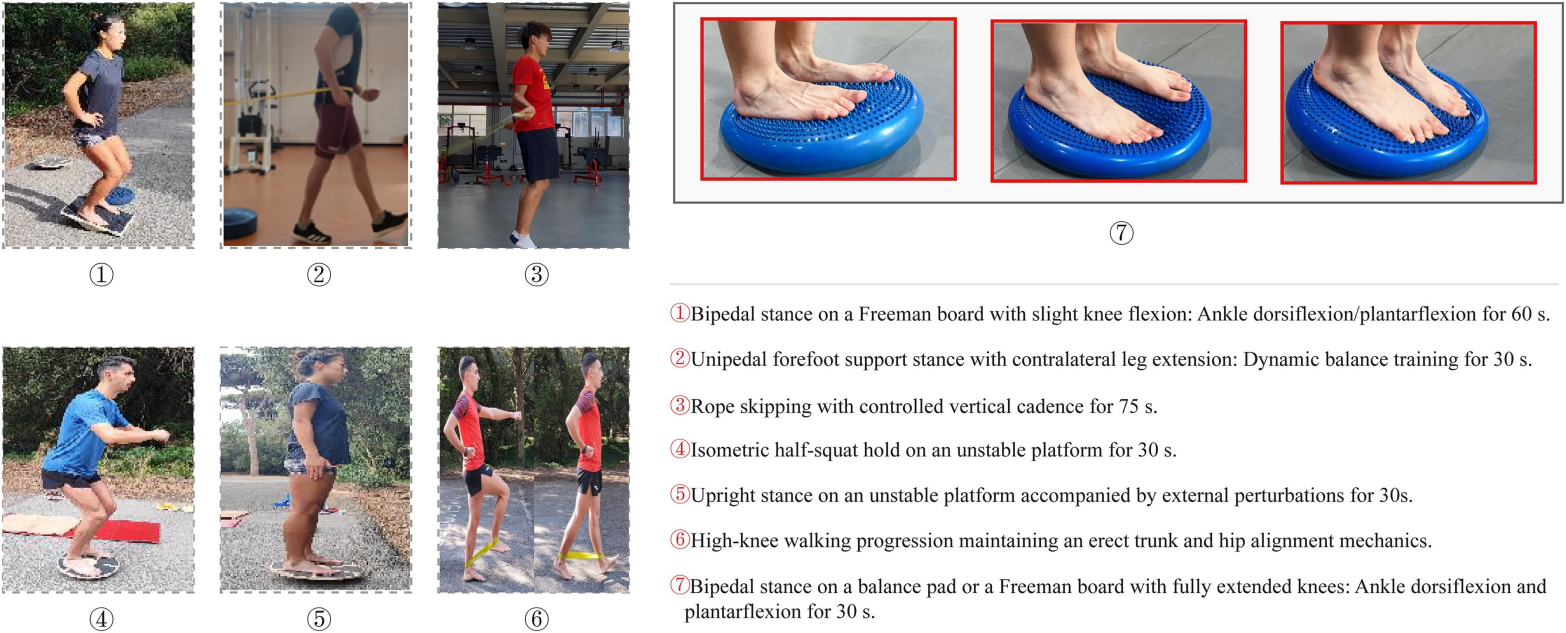
Proprioceptive training content. Representative proprioceptive training exercises used during the Paris 2024 Olympic preparation period. Panels ①–⑦ show selected exercises involving bipedal stance on a Freeman board with slight knee flexion, unipedal forefoot support with contralateral leg extension, rope skipping with controlled vertical cadence, an isometric half-squat hold on an unstable platform, upright stance on an unstable platform with external perturbation, dynamic high-knee walking, and bipedal stance with fully extended knees on a balance pad or a Freeman board, respectively. These exercises were incorporated to challenge ankle proprioception, postural control, single-leg stability, lower-limb coordination, and race-walking technical control under fatigue.

Proprioceptive training comprised seven representative exercises involving stable and unstable support conditions, unilateral stance, external perturbation, rhythmic jumping, and dynamic gait-related tasks, as shown in Figure 4. These exercises were closely aligned with the technical requirements of race walking, especially the need to maintain legal and efficient mechanics under fatigue. Balance and unstable-surface training research suggests that such work may improve balance-related performance and neuromuscular control when appropriately prescribed (36, 37).

### 3.7 Blood lactate responses across the preparation period

**Figure 5:**
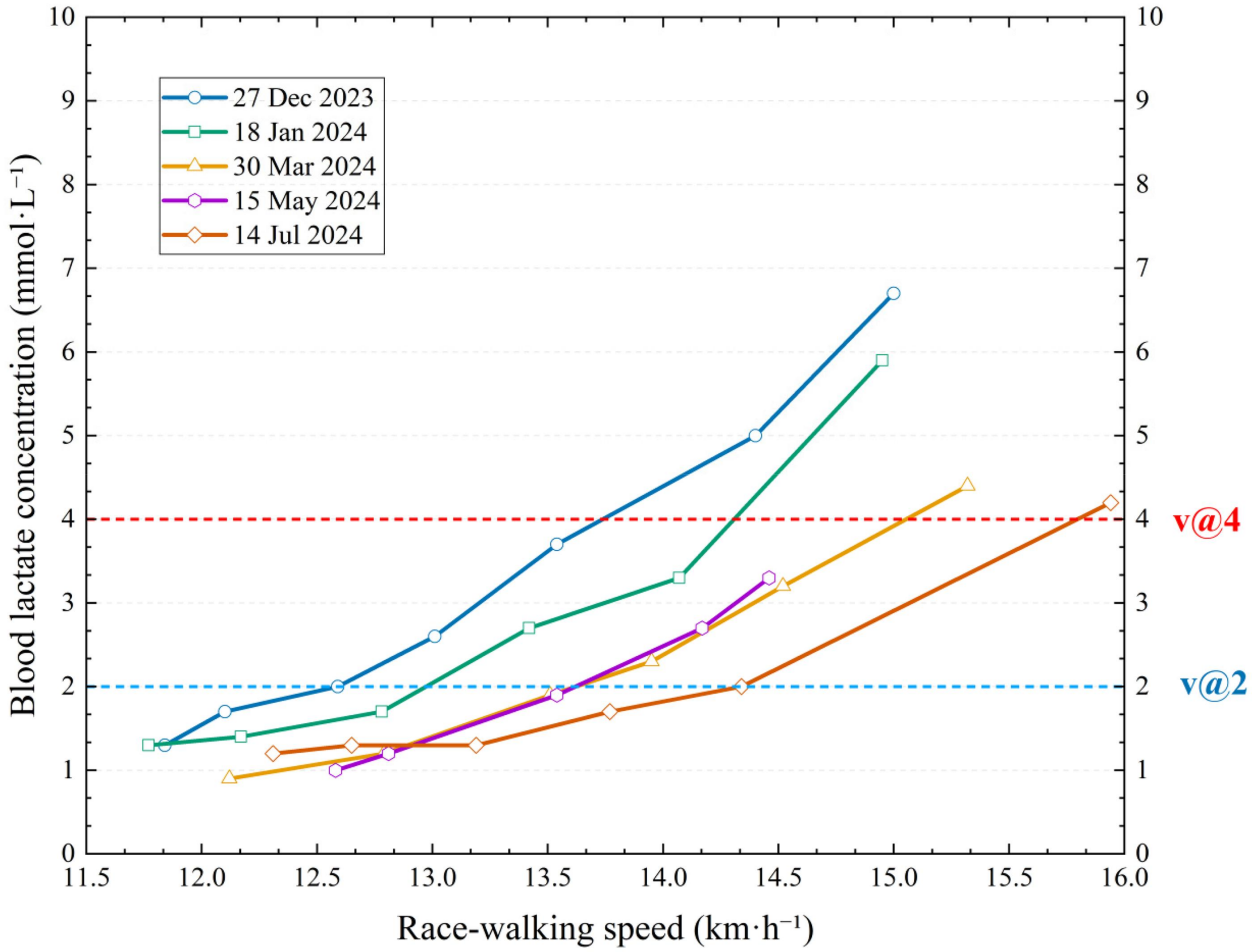
Blood lactate–speed relationships across the Paris 2024 Olympic preparation period. Blood lactate–speed relationships across the Paris 2024 Olympic preparation period. Blood lactate concentration was measured during incremental road-based race-walking tests conducted on 27 December 2023, 18 January 2024, 30 March 2024, 15 May 2024, and 14 July 2024. Capillary blood samples were collected from the earlobe immediately after each incremental stage. Race-walking speed was calculated from the recorded stage distance and completion time, except for the test conducted on 15 May 2024, for which speed values were obtained directly from the original physiological monitoring record. The horizontal dashed lines indicate the fixed operational reference concentrations of 2.0 and 4.0 mmol L^−1^ used for training-intensity classification and longitudinal comparison. These concentrations should not be interpreted as individually determined physiological thresholds such as LT1 or LT2. The speed at 4.0 mmol L^−1^ was not estimated for 15 May 2024 because the highest measured blood lactate concentration during that test was 3.3 mmol L^−1^ and no extrapolation beyond the measured range was performed.

Blood lactate responses were analyzed across different monitoring time points to describe changes in the relationship between lactate concentration and race-walking speed during the Paris 2024 Olympic preparation period. Lactate responses were recorded during routine incremental race-walking tests and matched with the corresponding race-walking speeds calculated from the actual completed stage distance and time. The measured blood lactate values at each incremental stage are presented in Table 2, the interpolated speeds at fixed lactate reference points are shown in Table 12, and the corresponding lactate–speed relationships are presented in Figure 5.

**Table 12:** Estimated race-walking speeds at fixed blood lactate reference points and longitudinal changes across the preparation period.

Date	Speed at 2 mmol L^−1^ (km h^−1^)	Pace (min km^−1^)	*Δ* speed (km h^−1^)	Change (%)	Speed at 3 mmol L^−1^ (km h^−1^)	Pace (min km^−1^)	Speed at 4 mmol L^−1^ (km h^−1^)	Pace (min km^−1^)	*Δ* speed (km h^−1^)	Change (%)	Speed difference, 2–4 mmol L^−1^ (km h^−1^)	Slope
27 Dec 2023	12.59	4:46	0	0.0	13.20	4:33	13.68	4:23	0	0.0	1.08	1.85
18 Jan 2024	12.97	4:38	0.38	3.0	13.74	4:22	14.31	4:12	0.63	4.6	1.34	1.50
30 Mar 2024	13.62	4:24	1.02	8.1	14.39	4:10	15.05	3:59	1.38	10.1	1.43	1.40
15 May 2024	13.62	4:24	1.02	8.1	14.32	4:11	—	—	—	—	—	—
14 Jul 2024	14.34	4:11	1.75	13.9	15.07	3:59	15.79	3:48	2.12	15.5	1.45	1.38

Speeds corresponding to blood lactate concentrations of 2.0, 3.0, and 4.0 mmol L^−1^ were estimated by linear interpolation between adjacent measured stages. Absolute and percentage changes were calculated relative to the first monitoring session on 27 December 2023. The speed difference was calculated as the speed at 4 mmol L^−1^ minus the speed at 2 mmol L^−1^. The slope was calculated as 2/(speed at 4 mmol L^−1^−speed at 2 mmol L^−1^) and is expressed as mmol L^−1^ (km h^−1^)^−1^. The speed at 4 mmol L^−1^ was not estimated for 15 May 2024 because the highest measured blood lactate concentration was 3.3 mmol L^−1^. An em dash indicates that the value could not be estimated.

Overall, the lactate–speed relationship showed a progressive rightward shift across the preparation period. The estimated speed at the fixed blood lactate concentration of 2.0 mmol L^−1^ increased from 12.59 km h^−1^ on 27 December 2023 to 12.97 km h^−1^ on 18 January 2024, 13.62 km h^−1^ on 30 March 2024, 13.62 km h^−1^ on 15 May 2024, and 14.34 km h^−1^ on 14 July 2024. Relative to the first monitoring session, the final monitoring session showed an increase of 1.75 km h^−1^, corresponding to a 13.9% increase. Thus, during the later preparation stages, a higher race-walking speed was associated with the same fixed blood lactate concentration of 2.0 mmol L^−1^.

The estimated speed at the fixed blood lactate concentration of 4.0 mmol L^−1^ also increased across the preparation period. It increased from 13.68 km h^−1^ on 27 December 2023 to 14.31 km h^−1^ on 18 January 2024, 15.05 km h^−1^ on 30 March 2024, and 15.79 km h^−1^ on 14 July 2024. Based on the unrounded interpolated values used in the calculations, the final monitoring session represented an increase of 2.12 km h^−1^, or 15.5%, relative to the first monitoring session. The speed at 4.0 mmol L^−1^ could not be estimated for the test conducted on 15 May 2024 because the highest measured blood lactate concentration during that session was 3.3 mmol L^−1^; therefore, no extrapolation beyond the measured range was performed.

The lactate–speed relationship also became flatter over the preparation period. The race-walking speed difference between the fixed blood lactate concentrations of 2.0 and 4.0 mmol L^−1^ increased from 1.08 km h^−1^ on 27 December 2023 to 1.34 km h^−1^ on 18 January 2024, 1.43 km h^−1^ on 30 March 2024, and 1.45 km h^−1^ on 14 July 2024. Correspondingly, the slope between 2.0 and 4.0 mmol L^−1^ decreased from 1.85 mmol L^−1^ (km h^−1^)^−1^ on 27 December 2023 to 1.50 on 18 January 2024, 1.40 on 30 March 2024, and 1.38 mmol L^−1^ (km h^−1^)^−1^ on 14 July 2024. These changes quantitatively describe a flatter lactate response profile across the monitoring period.

The date-specific lactate–speed relationships showed a broadly progressive pattern. On 27 December 2023, during the early winter preparation phase, blood lactate concentration increased relatively rapidly as race-walking speed increased. By 18 January 2024, higher speeds below approximately 14 km h^−1^ were associated with comparatively lower blood lactate concentrations. During the competition-specific intensification phase, the relationship shifted further rightward on 30 March 2024. On 15 May 2024, blood lactate concentration remained below 4.0 mmol L^−1^ throughout the test, and this monitoring session therefore primarily reflected responses within the lower and moderate concentration ranges. By 14 July 2024, the athlete achieved the highest estimated race-walking speeds at both 2.0 and 4.0 mmol L^−1^.

Taken together, the increase in estimated race-walking speed at fixed blood lactate concentrations and the reduction in the slope between 2.0 and 4.0 mmol L^−1^ indicate that comparable blood lactate concentrations were associated with higher race-walking speeds during the later monitoring sessions. Because the tests were conducted on outdoor roads as part of routine physiological monitoring and differed in stage configuration and interstage recovery duration, these findings should be interpreted as descriptive road-based monitoring indicators rather than as laboratory-derived or individually determined physiological threshold parameters.

### 3.8 Integrated training framework during Olympic preparation

**Figure 6:**
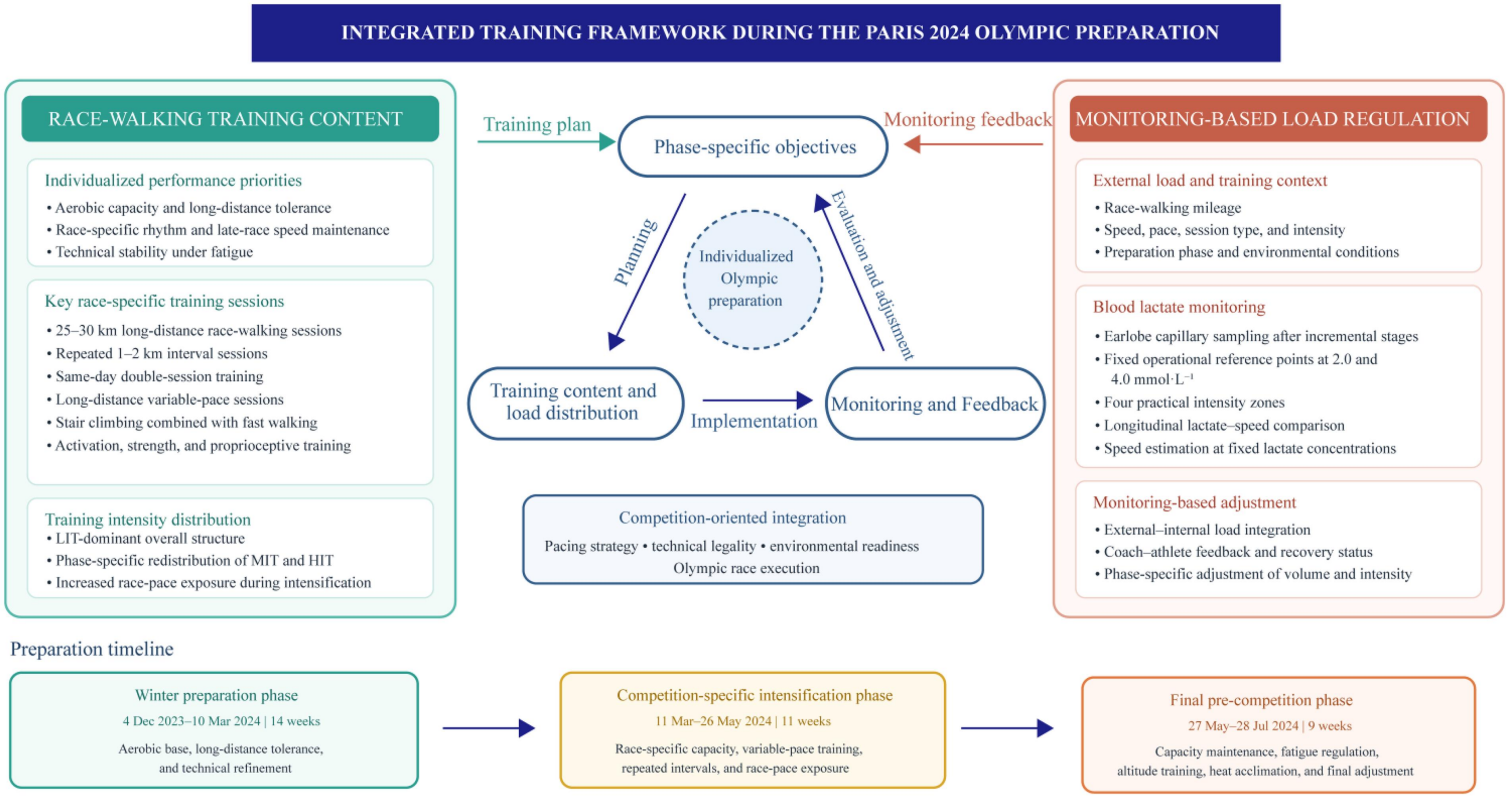
Integrated training framework for Jiayu Yang's Paris 2024 Olympic preparation. The framework links phase-specific objectives, race-walking training content and intensity distribution, and monitoring-based load regulation across the winter preparation, competition-specific intensification, and final pre-competition phases. Key race-specific sessions included 25–30 km long-distance race-walking sessions, repeated 1–2 km intervals, same-day double-session training, long-distance variable-pace sessions, stair climbing combined with fast walking, and complementary activation, strength, and proprioceptive training. These sessions were coordinated within a predominantly low-intensity training structure, with phase-specific redistribution of moderate- and high-intensity work. Race-walking mileage, speed, pace, session content, blood lactate responses, coach–athlete feedback, recovery status, and environmental preparation were integrated to support dynamic load adjustment and competition-oriented preparation. Fixed blood lactate concentrations were used as operational reference points rather than as individually determined physiological thresholds.

Based on the phase division, training intensity distribution, key race-specific sessions, lactate monitoring, and Olympic pacing execution described above, the integrated training model for Jiayu Yang's Paris 2024 Olympic preparation is summarized in Figure 6. The model shows that the preparation process was organized around three connected components: phase-specific training objectives, race-walking-specific training content, and monitoring-based load regulation. Aerobic accumulation provided the foundation for subsequent threshold-oriented and race-pace work, while key race-specific sessions were arranged to support endurance capacity, rhythm control, late-race speed maintenance, and technical stability under fatigue. Blood lactate monitoring was used to connect external pace prescription with internal physiological response, and the final competition strategy linked training preparation with Olympic pacing execution.

## 4 Discussion

### 4.1 Individualized periodization across the Olympic preparation cycle

The present longitudinal case study shows that the athlete's Paris 2024 Olympic preparation was organized through an individualized periodized structure in which training volume, intensity distribution, and race-specific content were adjusted according to phase-specific goals. This structure is consistent with the general logic of periodization in high-performance sport and with endurance-training evidence emphasizing the organization of training intensity across zones (7–11, 23, 31, 32).

The competition-specific intensification phase shifted the training emphasis from capacity accumulation to the conversion of established aerobic capacity into race-specific performance. Variable-pace long-distance sessions, repeated intervals, and race-pace work increased the exposure to threshold-oriented and race-specific intensities, which is consistent with evidence that the sequencing and distribution of training intensity can influence endurance adaptation and performance outcomes (23, 25–29, 32).

### 4.2 Overall and phase-specific training intensity distribution

The present case was characterized by an overall pyramidal training intensity distribution. Across the 8-month Olympic preparation period, low-intensity training accounted for 81.9% of total race-walking mileage, whereas threshold-oriented and race-pace intensity accounted for 9.4% and 8.7%, respectively. This profile was therefore more consistent with a pyramidal model than with a polarized or threshold-oriented model.

A pyramidal training intensity distribution is generally characterized by a substantial low-intensity training base, with lower proportions of moderate- and high-intensity work. Previous research indicates that the intensity distribution adopted by endurance athletes is influenced by event characteristics, preparation phase, training history, total training volume, and the approach used to define intensity domains. Accordingly, no single distribution should be regarded as a fixed template for all athletes or throughout an entire training year (9, 10). Filipas et al. (23) further reported that both pyramidal and polarized distributions may promote adaptation in trained endurance athletes, although their effects depend on the organization of the training cycle and the athlete's training background.

In the present case, the winter preparation and final pre-competition phases displayed relatively clear pyramidal structures. During winter preparation, the predominance of low-intensity work provided the volume base for long-distance race walking and subsequent race-specific training. During the final pre-competition phase, the continued predominance of low-intensity work served a different practical purpose, namely the maintenance of aerobic capacity, regulation of fatigue, and preservation of race-specific rhythm before competition. Previous endurance-training research suggests that a substantial low-intensity foundation may facilitate the accumulation of training volume and provide a basis for the selective introduction of more demanding race-specific stimuli (31, 32).

The competition-specific intensification phase did not show the strict classical ordering of LIT > MIT > HIT. Although low-intensity training remained dominant, accounting for 80.6% of phase mileage, race-pace intensity accounted for 13.2% and exceeded threshold-oriented training, which accounted for 6.2%. This phase should not be interpreted as a complete transition to a polarized distribution, because the overall structure remained strongly dominated by low-intensity training and the proportion of high-intensity work did not become disproportionately large. Rather, the data indicate a phase-specific redistribution of training intensity, in which total training volume was reduced and race-pace exposure was selectively increased to facilitate the conversion of established aerobic capacity into race-specific performance. This approach is consistent with the view that training intensity distribution should be adjusted dynamically according to the objectives of each preparation phase (10, 23).

Taken together, the athlete's preparation was not characterized by a fixed pyramidal template applied uniformly throughout the year. Instead, it reflected an individualized structure built on an overall pyramidal distribution, with phase-specific redistribution toward greater race-pace exposure during competition-specific intensification. For elite 20 km race walkers, training intensity distribution should therefore be adjusted dynamically in relation to event-specific speed demands, technical-stability requirements under fatigue, and the objectives of each preparation phase.

### 4.3 Lactate monitoring and training-load regulation

Blood lactate monitoring provided a practical bridge between external training load and internal physiological response. Because the lactate data were collected as part of routine training monitoring rather than under laboratory-controlled conditions, the results should be interpreted descriptively. Nevertheless, the rightward shift and flatter profile of the lactate–speed relationship observed across the preparation period are consistent with improved tolerance of higher race-walking speeds and enhanced lactate handling, which are commonly discussed in relation to endurance adaptation and training-intensity regulation (12–14, 38, 39).

This finding supports the practical value of combining pace-based training prescription with physiological monitoring in elite race walking. In a technically constrained endurance event, race-walking speed alone may not fully represent the internal load imposed on the athlete. Lactate monitoring can therefore help coaches evaluate whether a given pace is being completed under acceptable metabolic stress, whether threshold-oriented work is being appropriately prescribed, and whether phase-specific load adjustments are needed.

### 4.4 Competition strategy and performance-oriented integration

A further feature of this preparation process was the integration of training structure with the planned Olympic pacing strategy. The pre-competition plan emphasized a stable opening phase, a controlled middle phase, and a stronger final phase. Accordingly, the final pre-competition phase was organized to maintain race-specific rhythm, regulate fatigue, and preserve competition readiness through continued race-specific stimuli, progressive load adjustment, and environmental preparation.

This integration was reflected in the sequential organization of the final pre-competition phase. After a short pre-altitude preparation period, the athlete completed a moderate-altitude training block in Roccaraso, Italy, followed by heat acclimation and final competition adjustment in Lido di Ostia, Rome. This arrangement allowed race-specific training to be maintained while environmental exposure was incorporated into the broader preparation structure. Previous endurance-training research has shown that altitude exposure can be integrated into endurance preparation when coordinated with sport-specific training and appropriate load management (40, 41). Heat acclimation has also been recommended for athletes preparing for competition in hot and humid environments because of its relevance to thermoregulatory preparation and tolerance to environmental stress (42, 43).

This performance-oriented integration is important because elite race walking is not determined by endurance capacity alone. Athletes must sustain a high legal walking velocity while managing metabolic stress, technical judging demands, race dynamics, and environmental conditions. Therefore, the training structure should be interpreted as an integrated preparation system connecting physiological capacity, race-specific rhythm, environmental preparation, technical stability, and competition execution, rather than as a collection of isolated training methods (1–6, 24, 44).

### 4.5 Practical applications

Several practical implications can be drawn from this longitudinal case study. First, preparation for elite 20-km race walking may benefit from a clearly phased but flexible periodized structure. In the present case, the preparation process progressed from winter aerobic development and technical refinement to competition-specific intensification and, subsequently, final pre-competition regulation. The function of each phase was therefore defined not only by changes in total training volume, but also by the redistribution of intensity, the selection of race-specific sessions, and the integration of physiological and environmental monitoring. For coaches and support teams, this finding suggests that phase division should be based on the athlete's current training status, competition schedule, technical limitations, and race-specific demands rather than on a rigid calendar-based model. Training objectives, volume, intensity, and session content should be adjusted dynamically as the athlete moves from general capacity development toward competition readiness (7, 8).

Second, training intensity distribution should be interpreted in relation to both the overall preparation cycle and the specific objectives of each phase. Across the complete preparation period, low-intensity training accounted for 81.9% of total race-walking mileage, whereas threshold-oriented and race-pace training accounted for 9.4% and 8.7%, respectively. This pattern was broadly consistent with a pyramidal distribution. However, the competition-specific intensification phase showed a phase-specific redistribution in which race-pace mileage exceeded threshold-oriented mileage, while low-intensity training remained dominant. This indicates that the practical value of training intensity distribution lies not in reproducing a fixed pyramidal or polarized template, but in maintaining sufficient low-intensity volume while selectively increasing race-specific intensity when required. In elite race walking, the classification and interpretation of training intensity should therefore combine race-walking speed, blood lactate response, accumulated mileage, technical stability, recovery status, and competition objectives rather than relying solely on generic intensity-zone models (9, 10, 23).

Third, repeated blood lactate monitoring may provide useful support for pace prescription and training-load regulation in elite race walking. In the present case, the speeds corresponding to fixed blood lactate reference points increased across the preparation period, while the slope between 2.0 and 4.0 mmol L^−1^ decreased. These changes indicated that higher race-walking speeds were associated with comparable blood lactate concentrations during later monitoring sessions. In practical settings, coaches may use this type of longitudinal information to evaluate whether a prescribed pace imposes an appropriate internal metabolic demand, whether the athlete is adapting to threshold-oriented and race-pace training, and whether phase-specific load adjustment is required. However, such data should not be interpreted in isolation or treated as laboratory-derived physiological thresholds. Their principal value lies in repeated assessment under reasonably consistent road-based testing conditions and in combination with training records, race-walking speed, environmental context, and coach–athlete feedback (12–14).

Fourth, key race-specific training sessions should be assigned explicit functional roles within the periodized plan. In this case, 25–30 km long-distance race-walking sessions were mainly used to develop aerobic endurance, fatigue resistance, and pacing stability. Repeated 1–2 km intervals provided race-specific speed exposure and required the athlete to maintain velocity and technique under progressively increasing fatigue. Same-day double-session training increased cumulative race-walking load within a limited time period, whereas long-distance variable-pace sessions connected aerobic endurance with changes in rhythm and late-session acceleration. Stair climbing followed by fast walking combined supplementary lower-limb strength-endurance loading with subsequent race-specific work. The practical value of these sessions was therefore determined less by the isolated use of any single method than by their timing, intensity, recovery structure, and relationship to the objectives of the corresponding preparation phase. Coaches should avoid treating individual sessions as universally effective prescriptions and should instead consider how each session contributes to endurance development, race-specific rhythm, late-race speed maintenance, and technical stability under fatigue (25–27, 33).

Fifth, final competition preparation should integrate physiological capacity, pacing strategy, technical legality, environmental demands, and recovery regulation. The present case included a moderate-altitude training period followed by heat acclimation and final competition adjustment. These elements were not implemented as independent interventions but were incorporated into the broader preparation structure while race-specific training was maintained. For elite race walkers, this integrated approach is particularly important because performance depends on the ability to sustain a high legal walking velocity while responding to tactical changes, environmental stress, hydration demands, and accumulated fatigue. Accordingly, final preparation should connect training-load adjustment, race-pace exposure, technical monitoring, environmental adaptation, and planned pacing strategy within a coordinated support system rather than addressing these components separately (24, 43, 44).

Overall, the practical significance of this case lies in the coordinated organization of training rather than in any single training method or physiological indicator. The findings suggest that preparation for elite 20 km race walking may be strengthened by aligning phase-specific objectives, training intensity distribution, race-specific session design, blood lactate monitoring, environmental preparation, and competition strategy. Nevertheless, because the present evidence was derived from one Olympic champion in a real-world training environment, these practices should be adapted to the individual athlete's training history, physiological characteristics, technical profile, and competition context rather than transferred directly as a standardized model.

### 4.6 Limitations

This study has several limitations. First, it is a single-athlete case study, and the findings should not be generalized directly to all female race walkers. Second, because the study analyzed real-world training data rather than a controlled experimental intervention, causal relationships between specific training methods and Olympic performance cannot be established. Third, some physiological monitoring data were collected as part of routine national-team practice, and therefore the testing conditions were not as standardized as laboratory-based protocols. Fourth, due to the confidentiality requirements of elite sport, some tactical and physiological information could not be reported in full detail. Future research should combine longitudinal monitoring, standardized physiological testing, biomechanical analysis, and multi-athlete comparisons to further clarify the mechanisms of elite race-walking preparation (11, 12, 17, 20–22, 24, 44).

## 5 Conclusions

This longitudinal case study described the periodized training structure, training intensity distribution, key race-specific training sessions, competition-oriented pacing strategy, and blood lactate responses of a female Olympic champion 20 km race walker during preparation for the Paris 2024 Olympic Games. The preparation process was organized around three functional phases: winter preparation, competition-specific intensification, and final pre-competition phase. Across these phases, training progressed from high-volume aerobic development and technical refinement to race-specific intensity regulation, fatigue control, and performance expression before the Olympic competition.

The findings suggest that the athlete's Olympic preparation was characterized by an individualized and integrated training framework. High-volume, low- to moderate-intensity race walking provided the aerobic foundation for later race-specific work; long-distance variable-pace sessions, repeated intervals, same-day double-session training, hill training, and progressive acceleration sessions were used to develop speed endurance, late-race tolerance, and technical stability under fatigue. Blood lactate monitoring served as a practical tool for connecting training intensity classification, pace prescription, and load adjustment. The observed rightward shift and flatter profile of the lactate–speed relationship indicated that higher race-walking speeds were associated with comparable blood lactate concentrations during the later stages of preparation.

Overall, this study provides descriptive longitudinal evidence of how periodization, training intensity distribution, race-specific training content, blood lactate monitoring, and competition strategy were integrated during preparation for elite 20 km race walking. Because the study involved a single athlete in a real-world high-performance training environment, the findings should be interpreted as an applied case reference rather than as a universally generalizable training model. Nevertheless, this case offers practical insight for coaches and support teams seeking to organize individualized, systematically monitored, and competition-oriented preparation for elite endurance athletes.

## Data Availability

The original contributions presented in the study are included in the article/Supplementary Material, further inquiries can be directed to the corresponding author.
